# Supplementary material for: Prevalence, risk factors and health seeking behaviour of pulmonary tuberculosis in four tribal dominated districts of Odisha: Comparison with studies in other regions of India
Source: PLoS One. 2020 Apr 6;15(4):e0227083. doi: 10.1371/journal.pone.0227083 (PMC7135306; doi:10.1371/journal.pone.0227083)
Supplement: Attachment — Submitted filename: Response to Reviewers - PLOS ONE.doc [file pone.0227083.s002.doc]

22^nd^ Jan.,2020

**Response to Reviewers**

Dear Sir,

I thank you for reviewing the manuscript and giving me a chance to revise the same. I have incorporated the changes in the manuscript as suggested and highlighted in **blue.**

Thanking you & with regards,

Sincerely

Tahziba Hussain

Dr.Tahziba Hussain

1. Please ensure that your manuscript meets PLOS ONE's style requirements, including those for file naming. The PLOS ONE style templates can be found at http://www.plosone.org/attachments/PLOSOne_formatting_sample_main_body.pdf and <http://www.plosone.org/attachments/PLOSOne_formatting_sample_title_authors_affiliations. pdf>

**Ans. I have checked the manuscript for PLOS ONE's style requirements and made changes accordingly.**

2. Please include additional information regarding the survey or questionnaire used in the study and ensure that you have provided sufficient details that others could replicate the analyses.

Ans. **The details of the questionnaire have been included in both the local language, Odiya and English, as Supporting Information.**

3. Please amend either the title on the online submission form (via Edit Submission) or the title in the manuscript so that they are identical.

**Ans. The title has been amended. Comparison with studies in other regions of India has been added in the title in the online submission.**

 4. Your ethics statement must appear in the Methods section of your manuscript. If your ethics statement is written in any section besides the Methods, please move it to the Methods section and delete it from any other section.

**Ans. Ethics approval has been given in the Methodology section.**

5. We note that Figure #1 in your submission contains map images which may be copyrighted. All PLOS content is published under the Creative Commons Attribution License (CC BY 4.0), which means that the manuscript, images, and Supporting Information files will be freely available online, and any third party is permitted to access, download, copy, distribute, and use these materials in any way, even commercially, with proper attribution. For these reasons, we cannot publish previously copyrighted maps or satellite images created using proprietary data, such as Google software (Google Maps, Street View, and Earth). For more information, see our copyright guidelines: http://journals.plos.org/plosone/s/licenses-and-copyright.

We require you to either (a) present written permission from the copyright holder to publish these figures specifically under the CC BY 4.0 license, or (b) remove the figures from your submission:

**Ans. The figure 1 containing the map has been removed from the manuscript.**

1. There needs some clarification in the Abstract to help readers easily comprehend the points. Line 37, out of these (?). Line 38, these (?). Line 40, are these 18 culture-positive among those 35 TB-active? Line 41-44, I could not understand what these sentences mean? Line 45, the conclusion sentence dose not convey any insightful conclusion.

**Ans. Line 37 - 40, the sentences have been re-framed. In Line 41-44, the risk factors have been elaborated.** **Conclusion sentence has been changed.**

2. The Introduction section needs to be cleaned. It is better to get to the point directly. In the manuscript, however, a lengthy introduction of tribal structure in India lead the way, from line 53 through line 70. The logic chain from line 79-89 is not clear. Try to follow the general structure, for example, what is the aim, what is the expectation, what is the strategy and method.

**Ans. Line 79 - 89, the sentences have been re-framed including the aim, expectation, strategy and method.**
